# Do You Feel What I Feel? Parental Perception of Pain Intensities, Level of Suffering, and Pain Impairment in Chronic Pain in Children and Adolescents

**DOI:** 10.1002/brb3.70945

**Published:** 2025-09-30

**Authors:** Adam Geremek, Ishita Noori Haider, Martin Jung, Manuel Munz

**Affiliations:** ^1^ Psychosomatikum Outpatient Clinic Kiel Kiel Germany; ^2^ Outpatient Clinic of the German Association of Cognitive Behavioral Therapy Erlangen Erlangen Germany; ^3^ HELIOS Clinic for Child and Adolescent Psychiatry Schleswig Germany; ^4^ Clinic for Child and Adolescent Psychiatry Psychotherapy and Psychosomatics, Center for Integrative Psychiatry Kiel Germany; ^5^ Institute for Child and Adolescent Psychiatry Center for Integrative Psychiatry Kiel Germany

**Keywords:** children, chronic pain disorders, pain estimation, parental ratings

## Abstract

**Introduction:**

Ratings of pain intensities, level of suffering, and impairment through pain are important factors informing physicians and pain therapists with respect to therapy choice. This study aimed to investigate the concordance of children's and their parents’ ratings and the relationship of pain‐related measures with psychopathological variables.

**Methods:**

Children diagnosed with chronic pain disorders and comorbid psychiatric disorders (depression, anxiety, trauma‐, and stress‐related disorders according to ICD‐10; *N* = 152, *n* = 103 females and *n* = 49 males; mean age 15.5 years, age range 11–17 years) and their parents (*N* = 195, *n* = 120 mothers/*n* = 75 fathers) were assessed with ratings of mean and maximum pain level. Impairment through pain and level of suffering were assessed with respective questionnaires.

**Results:**

Fathers rated maximum pain lower than their children or mothers, while mothers rated the level of the child's pain suffering higher than their children or fathers. Both mothers and fathers underestimated the medium pain level compared to the children's assessment. We found intercorrelations of pain ratings and psychopathological measures.

**Conclusions:**

Discrepancies in pain‐related ratings in children with chronic pain and mental diagnoses indicate that the full picture of children's and both parents’ ratings should be considered for the adequate choice of therapeutic strategies and settings whenever possible.

## Graphical abstract

Because ratings of pain intensities and pain‐related impairments differ between children with chronic pain disorders and their caregivers, ratings of all the family members involved should be collected before treatment.

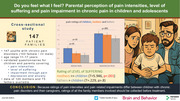

## 1 Introduction

Chronic pain is frequent in children (Chambers et al. 2024), having a huge impact on children's well‐being (Roth‐Isigkeit et al. 2005) and causing disability and distress (Huguet and Miró 2008), but also uncertainty (Tanna et al. 2020). During the past decades, there has been a considerable development of treatment options depending on the child's pain severity, disability, and comorbid emotional disorders (Yazdani and Zeltzer 2013; Fisher et al. 2019). For clinicians, the accurate assessment of pain intensities is important for the decision on adequate treatment settings. Since parents are crucial caregivers for children seeking medical and psychological support, the estimation of their child's pain is of great clinical importance. Depending on the child's age and mental or emotional condition, the parental assessment of the child's health condition may be the only indication available. However, the parental estimation may not be in accordance with their child's own pain experience (Sundblad et al. 2006). Up to now, findings on the agreement between child and parent assessments are conflicting, showing both underestimation of children's pain (Chambers et al. 1998;; Goubert et al. 2009) and also good matches (Lifland et al. 2018 Vetter et al. 2014). However, if the child's pain is underestimated, important treatments may not be carried out, which can lead to pain chronification. On the other hand, parental pain overestimation may translate into overprotection and dysfunctional parental behavior and bears an increased risk of unnecessary treatment with undesirable side effects (Harrison et al. 2020; Goubert et al. 2012). Generally, girls' pain gets frequently underestimated, while boys’ pain is often overestimated (Cohen et al. 2014). There are also reports on good matches between child and parent assessments, regardless of child gender (Lifland et al. 2018).

Chronic pain is also frequent in children with mental disorders (Mangerud et al. 2013;; Geremek et al. 2024). However, little is known about the accuracy of the assessment of pain intensity and subjective suffering of children and adolescents. Recently, Martin and colleagues (Martin et al. 2020) reported that discrepancies between parental and child reports of child anxiety and depressive symptoms may affect the association between child emotional symptoms and functional impairment. In this way, there may be differences in the pain assessment in children with and without emotional disorders like anxiety and depression. Addressing the concordance of children's and parents’ pain estimations and level of suffering, also in relation to emotional psychopathology, we investigated the highest and average pain and level of suffering estimations in children with chronic pain by ratings both of children and their parents while assessing depression and anxiety scores at the same time, allowing for examining interrelations with pain and level of suffering.

## 2 Patients and Methods

### 2.1 Study Design and Participants

The study was carried out between December 2015 and August 2021 at the child and adolescent inpatient and outpatient departments of the HELIOS Child and Adolescent Psychiatry Clinic in Schleswig, Germany. In this single‐center cohort study design, a total of 152 individuals aged between 11 and 17 and diagnosed with persistent somatoform pain disorder (F45.40) or chronic pain disorder with somatic and psychological factors (F45.41) according to ICD‐10 (WHO 2005) were identified. The study was approved by the local ethics committee in the State of Schleswig‐Holstein, Germany (placed at the local Medical Chamber in Bad Segeberg; AZ 057/11 (II)), amendment on July 30, 2015 of an approval on May 19, 2011, and was performed in accordance with the Declaration of Helsinki in 1964. The participants and their parents were informed about the study, and both written and verbal informed consent were obtained. Diagnoses were determined by experienced physicians and specialists in child and adolescent psychiatry. Patients were excluded if their pain experience was explained by any somatic disorder. Five individuals identified with the above‐mentioned diagnoses were excluded from the initial 152 because almost no data were obtained. Pain location and organ systems were collected, too, as well as comorbid diagnoses and anxiety and depression scores, in order to characterize the sample. During the first appointment, patients and their caregivers were directed to complete a series of validated questionnaires in child and parent versions covering chronic pain psychopathology, anxiety, and depression as described below. At the following appointment, questionnaires were returned. As such, data of 147 children, 120 mothers, and 75 fathers were available. For a subsample of 27 individuals (85.2% girls), data of the child and both parents are available. In 53 cases, complete data are available for children and mothers (73.6% girls).

### 2.2 Measures

#### 2.2.1 Pain Intensity: “German Pain Questionnaire for Children and Youths” (DSF‐KJ)

Experienced mean and maximum pain were assessed using the children and youths’ version and the parents’ version of the German Pain Questionnaire for Children and Adolescents (“Deutscher Schmerzfragebogen für Kinder und Jugendliche,” DSF‐KJ). Participants were asked to rate mean pain and maximum pain during the last 7 days on an 11‐point Likert scale ranging from 0 to 10 (0 = *no pain* to 10 = *hardest pain*). In a clinical context, pain intensities are divided as follows: 0 = *no pain*, 1–3 = *light pain*, 4–6 = *strong pain*, and 7–10 = *hardest pain* (Schroeder et al. 2010).

#### 2.2.2 Everyday Impairment Through Pain: Pediatric Pain Disability Index

The children's and youths’ as well as the parents’ versions of the P‐PDI were used to assess impairment in everyday life caused by pain. The P‐PDI consists of 12 items covering pain‐related general and physical impairments during the last 4 weeks prior to assessment in various areas of everyday life on a 5‐point Likert scale ranging from 1 = *never* to 5 = *always*. Responses were collapsed to a sum score, with results ranging from 12 = *no pain‐related impairment* to 60 = *strong pain‐related impairment*. Starting from a cut‐off value of 36, pain‐related impairment may be classified as “clinically relevant.” Internal consistency of the P‐PDI is acceptable (Cronbach's *α* = 0.87; Hübner et al. 2009).

#### 2.2.3 Level of Suffering: “Gießener Beschwerdefragebogen für Kinder und Jugendliche” (GBB‐KJ)

The GBB‐KJ was used to assess the level of suffering. Children and youths, as well as mothers and fathers, rated the frequency of 59 different physical complaints on a 5‐point Likert scale ranging from 0 = *never* to 4 = *continuously*. In this questionnaire, complaints are allocated to five different categories: (Chambers et al. 2024) exhaustion, (Roth‐Isigkeit et al. 2005) stomach trouble, (Huguet and Miró 2008) limb pain, (Tanna et al. 2020) circulation symptoms, and (Yazdani and Zeltzer 2013) symptoms of upper airway infection. According to the questionnaire manual, out of the 59 items, only 35 that are loading on the above‐mentioned category scales are used for further calculation. Raw value (maximum 140) can be transformed to *t* values according to age norms. *t* values range from 20 to 80, with values of 60 or higher meaning clinically significant suffering (Barkmann et al. 2008).

#### 2.2.4 Depressive Symptoms

In order to assess depressive symptomatology, the Depression Inventory for Children and Adolescents (DICA, Stiensmeier‐Pelster et al. 2014) was used. This is a validated, well‐established, and commonly used German self‐report questionnaire to quantitatively measure the grade of depression among children and adolescents. It consists of 26 items (e.g., “not liking oneself,” “loss of interest,” etc.), and the symptom presence is reported on a 4‐point Likert scale, ranging from 0 (“no symptom”) to 3 (“strong symptom”). German normative scores are available, and the results are reported in *t* values with *t* ≥ 60 being pathological.

#### 2.2.5 Anxiety Symptoms

We used the anxiety section of the Diagnostic System of Psychiatric Syndromes in Children and Adolescents (DISYPS‐CA; Döpfner et al. 2017), which is a validated and widely used German diagnostic tool for the assessment of self‐reported anxiety symptoms. Besides a general anxiety score (DISYPS anxiety), it includes subscales for social phobia, separation anxiety, and anxiety alertness. It consists of 32 items, and the symptoms are reported on a 4‐point Likert scale, ranging from 0 (“not at all”) to 3 (“very much”). Results are reported in stanines.

### 2.3 Data Analyses

Statistical analyses were performed using IBM SPSS Statistics, version 29, for Windows (IBM Corporation, Armonk, NY, USA). Descriptive statistics were conducted to assess the prevalence of pain loci and comorbid diagnoses according to ICD‐10 (WHO 2004). We conducted repeated measure analyses of variance (rmANOVAs) for mean and maximum pain (Schroeder et al. 2010), impairment through pain (Hübner et al. 2009), and level of suffering (Barkmann et al. 2008), and with the within‐subject factor INFORMANT (child; mother; father). ANOVAs were decomposed with post hoc pairwise *t*‐tests. Experience of mean and maximum pain as well as level of suffering were not normally distributed in the ratings of children and both parents (Kolmogorov–Smirnov test: *p* < 0.02). However, with samples with *n* > 25 in each group, analyses of variance (ANOVA) can be considered as robust (Schmider et al. 2010). Secondarily, with respect to violation of normal distribution, we also calculated the nonparametric test, the Friedman test.

## 3 Results

### 3.1 Sample Characteristics

Among the 147 patients included in the study, 103 were female and 44 were male. The most frequent comorbid disorders according to ICD‐10 (WHO 2005) were emotional disorders (“F93.‐”; *n* = 66), depressive disorders (“F32.‐”; *n* = 27), trauma‐ and stress‐related disorders (“F43.‐”; *n* = 12), and anxiety disorders (“F40.‐”; *n* = 10). More than one comorbid diagnosis was present in *n* = 21 individuals. Mean age was *M* = 15.50 years (SE = 0.13), with no age difference between female (*M* = 15.56, SE = 0.15 years) and male patients (*M* = 15.36, SE = 0.27 years; *F* = 0.519, *p* > 0.4). Gender, age, depression, and anxiety were exploratively compared among five locations of pain as assessed by the DSF‐KJ (Schroeder et al. 2010), using a 2 × 5 ANOVA with the between‐factors GENDER (female, male) and LOCATION (headache, migraine, stomachache, musculoskeletal pain, and multiple pain locations). Main effects of GENDER, LOCATION, and GENDER × LOCATION interactions for age, depression score, and anxiety score are shown in Table 1. There was a main effect of GENDER for depression score corresponding to gender comparisons (*F* = 5.625; *p* = 0.019), but no other effects of GENDER or LOCATION were found for age or depression scores (*p *> 0.1). However, there was a main effect of LOCATION for anxiety score [*F*(4) = 3.086, *p* = 0.018], but no effect of GENDER or GENDER × LOCATION interaction (*p* > 0.4). Post hoc *t*‐tests revealed that the main effect of GENDER for depression was due to female patients showing higher scores (*M* = 62.37, SE = 1.0) than male patients (*M* = 55.45, SE = 1.5; *F* = 14.938, *p* < 0.001), and the main effect of LOCATION for anxiety was due to patients with musculoskeletal pain showing higher anxiety scores (*M* = 8.29, SE = 0.24) than headache patients (*M* = 6.39, SE = 0.33; *p* = 0.003) and patients with multiple pain locations (*M* = 7.20, SE = 0.26; *p* = 0.042).

**TABLE 1:** Demografic data, diagnoses, depression and anxiety scores and pain locations.

	Pain location								
	headache/tension headache (*n* = 34)	migraine (*n* = 12)	stomachache (*n* = 27)	musculoskeletal pain (*n* = 21)	multiple pain locations (*n* = 53)	total (*N* = 147)	Gender	Location	Gender × Location
Gender (f/m)	28/6	6/6	17/10	18/3	34/19	103/44			
Age	15.39 (0.31)	15.58 (0.37)	15.29 (0.28)	15.32 (0.32)	15.73 (0.21)	15.50 (0.13)	*F* = 0.36; *p* > 0.8	*F* = 1.940, *p* > 0.5	*F* = 1.744, *p* > 0.1
Depression score (DICA, *t*‐score)	58.28 (2.00)	58.45 (2.66)	60.79 (1.63)	51.58 (2.29)	60.90 (1.53)	60.21 (0.87)	***F* = 5.625; *p* = 0.019**	*F* = 0.691, *p* > 0.6	*F* = 1.715, *p* > 0.15
Anxiety score (DISYPS‐anxiety, STANINE score)	6.39 (0.33)	7.18 (0.38)	7.09 (0.36)	8.29 (0.24)	7.20 (0.27)	7.13 (0.26)	***F* = 1.864, *p* = 0.027**	***F* = 3.086, *p* = 0.018**	*F* = 0.997, *p* > 0.4

### 3.2 Pain Intensities: Experience of Mean and Maximum Pain (DSF‐KJ)

Using an rmANOVA, there was no effect of INFORMANT [*F*(2) = 0.477, *p* = 0.091] for mean experienced pain. Post hoc *t* tests revealed that, compared to their children (*M* = 5.47, SE = 0.18), both mothers (*M* = 4.97, SE = 0.19; *T* = 2.267, *p* = 0.025) and fathers (*M* = 4.87, SE = 0.233; *T* = 2.348, *p* = 0.022) rated the mean experienced pain lower. Introducing GENDER (male, female) as an additional between‐subject factor revealed no effect of GENDER [*F*(1) = 1.403, *p* = 0.241], INFORMANT [*F*(2) = 1.517, *p* = 0.241], or GENDER × INFORMANT interaction (*p* > 0.3). Considering that experienced mean pain was not normally distributed, we also calculated the nonparametric test, the Friedmann test, showing no effect of INFORMANT [*F*(2) = 3.169, *p* = 0.21].

**FIGURE 1:**
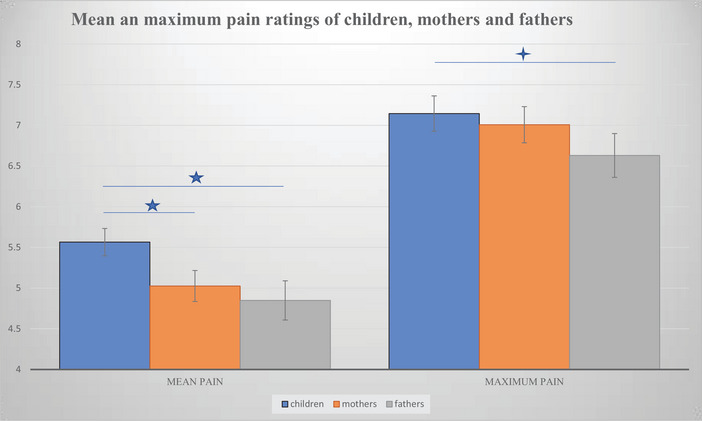
Means and standard errors of mean and maximum pain ratings of children, mothers and fathers Mean and maximum pain as rated by children (blue), mothers (yellow) and fathers (gray); *p* < 0.05 *p* < 0.001.

For maximum pain, rmANOVA revealed a highly significant effect of INFORMANT [*F*(2) = 12.423, *p* < 0.001]. The rmANOVA was decomposed with post hoc *t*‐tests showing no mean difference between children (*M* = 7.09, SE = 0.25) and mothers (*M* = 6.97, SE = 0.233; *T* = 0.609, *p* = 0.5; *n* = 110). However, we found significantly lower ratings of fathers (*M* = 6.65, SE = 0.26) compared to their children (*M* = 7.67, SE = 0.241; *T* = 4.329, *p* < 0.01; *n* = 62). Introducing GENDER (male, female) as an additional between‐subject factor revealed no effect of GENDER [*F*(1) = 1.980, *p* = 0.165], but the effect of INFORMANT remained significant [*F*(2) = 6.253, *p* = 0.032] with no GENDER × INFORMANT interaction (*p* = 0.270). Considering that maximum pain was also not normally distributed, we likewise calculated the nonparametric test, the Friedmann test, showing an effect of INFORMANT [*F*(9) = 8.934, *p* = 0.011]. Subsequent pairwise tests for paired samples revealed that this was due to fathers’ maximum pain rating being lower than their children's ratings (*T* = 2.742; *p* = 0.006). Ratings of pain intensities are depicted in Figure 1.

### 3.3 Everyday Impairment Through Pain (PDI)

rmANOVA showed no effect of INFORMANT [*F*(2) = 1.835, *p* = 0.164]. Introducing GENDER as a between‐subject factor revealed no effect of GENDER (*p* > 0.9), or INFORMANT (*p* > 0.4), but an INFORMANT × GENDER interaction [*F*(1,2) = 3.222, *p* = 0.044].

### 3.4 Level of Suffering (GBB‐KJ)

rmANOVA revealed a main effect of INFORMANT [*F*(2) = 6.642, *p* = 0.002]. Post hoc *t*‐tests revealed that mothers (*M* = 66.74, SE = 0.82) rated level of suffering higher than their children (*M* = 61.75, SE = 0.86; *T* = 5.946, *p* < 0.001; *n* = 117), while there was no difference in ratings of fathers (*M* = 62.22, SE = 1.21) and children (*M* = 61.92, SE = 0.95; *T* = 0.229, *p* < 0.8, *n* = 73). Introducing GENDER as a between‐subject factor revealed no effect of GENDER (*p* > 0.4), but the effect of INFORMANT remained significant [*F*(2) = 4.067, *p* = 0.019] with no GENDER × INFORMANT interaction (*p* = 0.362). Considering GBB‐KJ scores were not normally distributed, we also calculated the nonparametric test Friedmann test, also showing a significant effect of INFORMANT [*F*(2) = 24.261, *p* < 0.001], based on higher ratings of mothers compared to children (*F* = 4.198, *p* > 0.001) and fathers (*F* = 4.002, *p* > 0.001), while there was no difference between children and fathers (*p* > 0.8).

### 3.5 Correlation Analyses

Explorative Pearson's correlational analyses covering mean and maximum pain in relation to depression (DICA) and anxiety scores (DISYPS anxiety) revealed that mean and maximum pain were highly intercorrelated (*r* = 0.373, *p* < 0.001) as well as depression and anxiety scores (*r* = 0.680, *p* < 0.001). Further analyses showed that the mean pain and depression scores were just significantly correlated (*r* = 0.175; *p* = 0.049). No other pain and anxiety or depression correlation was found (*p* > 0.2). Mean pain was further correlated with level of suffering (GBB‐KJ) as rated by children themselves and fathers (children: *r* = 0.244, *p* = 0.005 and their fathers: *r* = 0.318, *p* = 0.01; mothers: *r* = 0.189; *p* > 0.05) and impairment through pain, also in children together with fathers (children: *r* = 0.289, *r* < 0.001; fathers: *r* = 0.246, *p* = 0.048) but not mothers (*r* = 0.071, *p* > 0.4), while maximum pain did not correlate with GBB‐KJ or P‐PDI^20^ scores for any of the informants (*p* > 0.01). Level of suffering (GBB‐KJ) was highly correlated with depression in children (DICA; *r* = 0.395, *p* < 0.001) and with mothers’ GBB‐KJ ratings (*r* = 0.235, *p* = 0.013) but not in fathers’ GBB‐KJ ratings (*r* = 0.207, *p* = 0.089), while anxiety (DISYPS anxiety) was highly correlated with level of suffering ratings in children (*r* = 0.442, *p* > 0.001) but neither with mothers’ (*r* = 0.131, *p* = 0.18) nor fathers’ ratings (*r* = 0.054, *p* < 0.6).

## 4 Discussion

In the present study, we examined perception of pain intensities, impairment through pain, and level of suffering in a selective child and adolescent psychiatric sample of children and youths with persistent or chronic pain. Representative of a systemic view, we collected ratings of both children and parents. We found diverging patterns of ratings of highest pain depending on whether fathers or mothers responded the questionnaires, irrespective of the children's gender: while fathers differed from mothers and rated maximum pain lower than their child or the corresponding mother, mothers rated the level of their child's pain suffering higher than their respective child or fathers, correspondingly. Statistically less significant, both mothers and fathers underestimated the mean pain level compared to the children's own estimation. Furthermore, the level of pain disability was rated equally by children and their parents. Moreover, we found that mean pain was associated with depressive symptomatology and correlated well with the level of suffering and impairment through pain in children and, somewhat surprisingly, in the father's assessments.

The literature on child–parent pain rating concordance is heterogeneous: in a pediatric population in acute postsurgical pain measures in older children, a good concordance is reported (Lifland et al. 2018) in children aged 8–18 years. This also holds true for child–parent reports in children without health distress (Waters et al. 2003). However, in younger children (children aged 4–7 years; Singer et al. 2002), with higher pain levels (Kelly et al. 2002), and in chronic pain (Sundblad et al. 2006), the pain ratings tend to become less accurate, and the child‐proxy agreement on the child's pain level is moderate to poor. These findings may suggest that pain ratings in conditions with obvious symptoms, such as injuries and acute pain, probably with functional parent–child relationships, are easier to rate than chronic disorders lasting for months or years, especially when chronic pain interferes with emotional symptoms and translates to parent–child interaction. Chronic pain conditions in children and adolescents, however, are closely associated with emotional symptoms and psychiatric disorders (Dorn et al. 2003 Skrove et al. 2015;; Mangerud et al. 2013 Geremek et al. 2024). In this way, our results are in line with prior findings, as mean pain in patients in our study correlated with the level of depression on the one hand, and as mothers and fathers in our study both underestimated mean pain ratings of their children and fathers more so the maximum pain on the other hand. The exact pathway of pain rating differences remains elusive. We propose that in acute pain, different cues, such as facial expression, motor behavior, and verbal expressions and their change from a healthy state, may be used by parents for the pain severity estimation (Kelly et al. 2002). In mentally affected children, these cues may diminish or change in depressive, school‐absent, and socially withdrawn children, so pain estimation of their children might become more difficult for parents.

Maximum pain levels were underestimated by fathers only. This may be due to fathers spending less time with the child affected by pain, as opposed to mothers on average. Thus, mothers more often witness pain exacerbations when children refuse to go to school or return from school earlier due to pain. However, if consequences of the pain conditions become visible (constant school absenteeism and social withdrawal), fathers and mothers equally consent in their estimations, as we found in a good child–parent agreement on the children's pain‐related disability. And even if the child–parent ratings correlated well, this is not equivalent to correctness: in a study with children with chronic pain (Hübner et al. 2009), pain‐related disability was correlated between children and their proxies. However, in this study, 57% of the parents systematically underestimated the children's ratings of their pain‐related disability, and in only 13% the child‐proxy ratings were corresponding. Interestingly, while in mentally healthy children there is a strong gender bias on the proxy estimation of the level of (acute) pain (Cohen et al. 2014), with girls’ pain being underestimated compared to the estimation of the same procedure in boys, we, in contrast, did not find any gender effect in our study. This may indicate that a gender effect is only present in children not or less affected mentally. Furthermore, our finding that mothers overestimated their children's level of suffering may be of special interest: children suffering from chronic pain often find it difficult to attend school due to their pain, causing distress to both parents and children. When they fail to attend school completely or are given a sick note by a physician, children's level of suffering might decline—while for parents, especially mothers, school absenteeism is the ultimate evidence of the child's disease—and consequently their estimation of the child's suffering increases. This may be driven by empathic distress, which is correlated with higher catastrophizing and overprotective behavior, which in turn is associated with poorer child functioning and higher child pain‐related impairment (Timmers et al. 2024).

xFinally, our findings of diverging estimations between fathers and mothers may indicate decisive differences in male and female approaches to observed pain: while pain disability was rated equally in children by their parents, pain suffering was overestimated by the mothers, while fathers’ assessment of mean pain correlated well with the child's pain impairment and pain suffering. This is in line with fathers’ less catastrophizing (Hechler et al. 2011), particularly rumination, and an important reference to clinicians that fathers may indeed underestimate their child's pain, but that their assessments of the overall condition of their child may be closer to the child's pain ratings. In this way, not only mothers but also fathers of children with chronic pain, particularly in children with comorbid mental disorders, may be important informants and targets for the child's pain therapist.

Given the highly selective sample, our results should be interpreted with caution. Also, we did not include a healthy control group in order to delineate healthy behavior from families with children with chronic pain. Notably, and corresponding to our limited resources, but also characteristic of diagnostic procedures in child and adolescent psychiatry, we did not obtain data from all questionnaires from all informants, which should be strived for in future studies. Putatively due to the sample being highly selective, a subset of our variables was not normally distributed. However, given the sample sizes, we still used parametric models to examine the effects of the informant, verifying each significant result with a nonparametric test. All of the significant differences we are reporting would also survive a Bonferroni correction for multiple testing. While our sample is rather selective as explained above, the sample size (*N* = 147) and the conclusiveness of including almost all of the families treated for these specific diagnoses are significant strengths of this study.

## 5 Conclusions

In contrast to findings in acute pediatric pain (Lifland et al. 2018) or absence of mental diagnoses (Waters et al. 2003), we found significant differences in the ratings of pain intensity, impairment, and level of suffering between the children and their parents in children with chronic pain. While fathers tend to rate pain intensities lower than mothers and children, mothers appear to overestimate the level of suffering as opposed to children and fathers. Our results both confirm and extend findings of gender‐specific parental perception of pain in their children (Cohen et al. 2014). From our findings, we cannot claim that integration of ratings by parents might allow for more reliable estimations of pain (intensities) but might instead provide a systemic view on pain perception. In particular, fathers’ perspectives on their children may provide crucial information and should therefore be taken into consideration when planning therapeutic interventions, which should cover parental and interactional aspects, too. Thus, our findings might encourage professionals in the field of pediatric pain therapy—especially in children with comorbid mental diagnoses—to consider parents’ and children's assessments equivalently in their therapy choice. Furthermore, future research might target underlying explanatory hypotheses of parental perception of pain and level of suffering depending on cultural aspects, traditional beliefs on pain, and their specific role, for example, stay‐at‐home parent or higher absence related to work life.

## Author Contributions

**Adam Geremek**: conceptualization, investigation, formal analysis, fund acquisition, writing – original draft. **Ishita Noori Haider**: investigation, data curation. **Martin Jung**: project administration, fund acquisition. **Manuel Munz**: Conceptualization, formal analysis, supervision, writing – review and editing

## Ethics Statement

The study was approved by the local ethics committee in Schleswig‐Holstein, Germany (placed at the local Medical Chamber in Bad Segeberg; AZ 57/11 (II)), approval on July 30, 2015, and was performed in accordance with the Declaration of Helsinki in 1964.

## Conflicts of Interest

The authors declare no conflicts of interest.

## Peer Review

The peer review history for this article is available at https://publons.com/publon/10.1002/brb3.70945.

## Data Availability

The data that support the findings of this study are available from the first author Adam Geremek, upon reasonable request
